# Comparative analysis of spreading depolarizations in brain slices exposed to osmotic or metabolic stress

**DOI:** 10.1186/s12868-021-00637-0

**Published:** 2021-05-03

**Authors:** Rita Frank, Ferenc Bari, Ákos Menyhárt, Eszter Farkas

**Affiliations:** Department of Medical Physics and Informatics, Faculty of Medicine and Faculty of Science and Informatics, University of Szeged, Korányi fasor 9, 6720 Szeged, Hungary

**Keywords:** Brain slice, Cerebral ischemia, Spreading depolarization, Osmotic stress, Oxygen‐glucose deprivation

## Abstract

**Background:**

Recurrent spreading depolarizations (SDs) occur in stroke and traumatic brain injury and are considered as a hallmark of injury progression. The complexity of conditions associated with SD in the living brain encouraged researchers to study SD in live brain slice preparations, yet methodological differences among laboratories complicate integrative data interpretation. Here we provide a comparative evaluation of SD evolution in live brain slices, in response to selected SD triggers and in various media, under otherwise standardized experimental conditions.

**Methods:**

Rat live coronal brain slices (350 μm) were prepared (n = 51). Hypo-osmotic medium (Na^+^ content reduced from 130 to 60 mM, HM) or oxygen-glucose deprivation (OGD) were applied to cause osmotic or ischemic challenge. Brain slices superfused with artificial cerebrospinal fluid (aCSF) served as control. SDs were evoked in the control condition with pressure injection of KCl or electric stimulation. Local field potential (LFP) was recorded via an intracortical glass capillary electrode, or intrinsic optical signal imaging was conducted at white light illumination to characterize SDs. TTC and hematoxylin-eosin staining were used to assess tissue damage.

**Results:**

Severe osmotic stress or OGD provoked a spontaneous SD. In contrast with SDs triggered in aCSF, these spontaneous depolarizations were characterized by incomplete repolarization and prolonged duration. Further, cortical SDs under HM or OGD propagated over the entire cortex and occassionally invaded the striatum, while SDs in aCSF covered a significantly smaller cortical area before coming to a halt, and never spread to the striatum. SDs in HM displayed the greatest amplitude and the most rapid propagation velocity. Finally, spontaneous SD in HM and especially under OGD was followed by tissue injury.

**Conclusions:**

While the failure of Na^+^/K^+^ ATP-ase is thought to impair tissue recovery from OGD-related SD, the tissue swelling-related hyper excitability and the exhaustion of astrocyte buffering capacity are suggested to promote SD evolution under osmotic stress. In contrast with OGD, SD propagating under hypo-osmotic condition is not terminal, yet it is associated with irreversible tissue injury. Further investigation is required to understand the mechanistic similarities or differences between the evolution of SDs spontaneously occurring in HM and under OGD.

## Background

In traumatic brain injury, stroke and anoxic brain injury caused by cardiac arrest, spreading depolarization (SD) plays a central pathophysiological role [1]. SD is also implicated in specific chronic neurological disorders including migraine with aura [2] or sudden unexpected death in epilepsy (SUDEP) [3]. SD sets off in the cerebral gray matter from a focal, critical tissue volume depolarizing synchronously (~ 1 mm^3^) [4], and is thought to be ignited by Na^+^/K^+^ ATPase failure [5] or neuronal hyperexcitability [6]. SD then propagates radially, or along anatomical (i.e. sulci in gyrencephalic brains) or functional barriers (i.e. area undergoing seizure) at a rate of 2–6 mm/min [7–9]. Over its course of propagation, SD is accompanied by a transient spreading depression of activity [10], tissue acidosis [11], cytotoxic edema [12], and a characteristic cerebral blood flow (CBF) response [13]. SD has been proposed to exacerbate ischemic or traumatic brain injury by deepening the metabolic crisis of tissue at risk [14, 15], and to correlate with unfavorable neurological outcome in these states [16–18]. In optimally perfused tissue, SD may not cause direct, gross injury [19], although SD has been implicated in the activation of trigeminal circuits and the evolution of migraine headache [20, 21], and the suspension of cardiorespiratory pacemaking in the brain stem in transgenic mouse models of SUDEP [3, 22].

The complexity and heterogeneity of conditions associated with SD in the living brain encouraged researchers to study SD in less complex model systems. In vitro live brain slice preparations have been suitable to recapitulate the neurophysiological hallmarks of SD accurately [23], and offer the opportunity to explore these changes free of cerebrovascular interferents [24]. The latter feature is especially pertinent when conducting optical imaging. Further, manipulations of the composition of the medium over the preparation allows the controlled simulation of global cerebral ischemia (e.g. oxygen glucose deprivation, OGD) [24], tissue acidosis (e.g. changing the bicarbonate or CO_2_ content of the medium) [25], or tissue swelling (e.g. hypo-osmotic medium, HM) [26]. Also, the application of pharmacological agents may yield highly reproducible, consistent data due to the uniform conditions, to aid the mechanistic understanding of SD evolution. For example, specific ion channels, exchangers or transporters mediating ion currents or amino acid translocations with SD may be studied effectively [27–31]. Finally, brain slice preparations are an ideal, basic experimental model to test the efficacy of SD inhibiting agents (e.g. NMDA or sigma-1 receptor blockers) with the goal to achieve neuroprotection [32, 33].

Even though a significant advantage of experimenting on live brain slices is the expected consistency of the results, there are methodological differences among laboratories, which complicate the integrative interpretation of data. The anatomical regions studied (cortex, hippocampus, striatum, subcortical gray matter or brain stem), the mode of SD initiation (e.g. high concentration KCl focal or global application, electric stimulation, transient hypoxia) or the type of the brain slice chamber used (interfaced or submerged preparations) may all modulate SD features in a manner specific for the given experimental approach [34].

Here we set out to provide a comparative evaluation of SD evolution in live brain slices, in response to selected SD triggers and in various media, under otherwise standardized experimental conditions. Local field potential (LFP) and intrinsic optical signal (IOS) imaging were applied for the assessment of the temporal and spatial characteristics of SD. This is the first study to explore the electrophysiological together with the intrinsic optical signal features of SDs under global oxygen-glucose deprivation or osmotic stress with respect to the physiological condition. The histological consequences of SD under these states are also evaluated. We pay particular attention here to spatial SD features, such as the volume of the area engaged in SD, which then corresponds to injury progression in the brain slice [31]. Rather than examining SD at the cellular level in a restricted tissue volume on the µm-scale [34, 35], we provide large scale (mm) whole slice measurements. The results are expected to aid the reliable reproduction of SD in live brain slice preparations, to serve the formulation of integrative conclusions.

## Results

### Prolonged SDs occur upon acute osmotic stress and in response to oxygen glucose deprivation

**Fig. 1:**
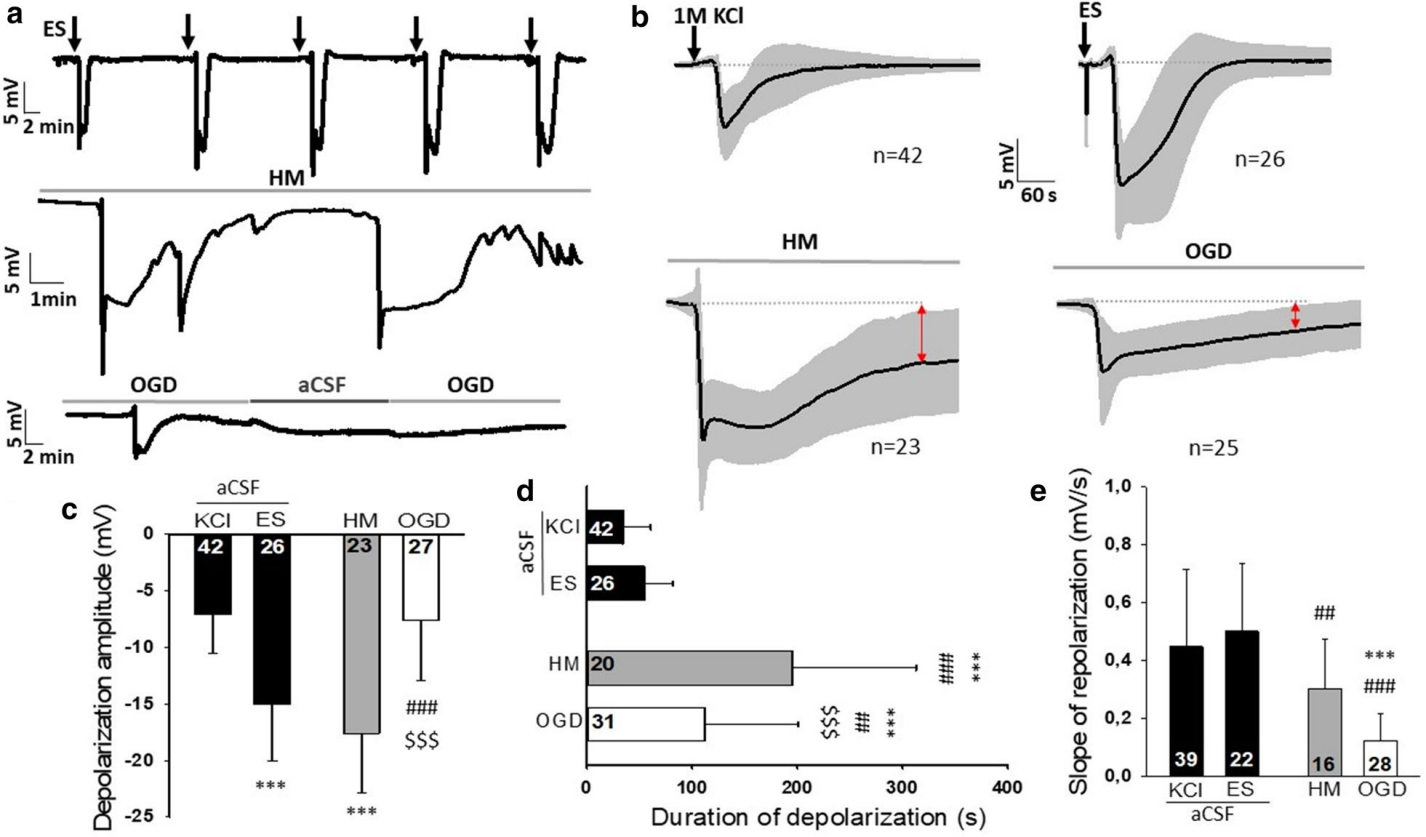
Electrophysiological characterization of spreading depolarizations.** a** Representative DC potential recordings demonstrate the experimental protocols. Slices were exposed to aCSF (SD initiation here: ES), HM, or OGD. Black vertical arrows indicate ES in aCSF, gray horizontal timelines depict the exposure to the experimental condition (HM or OGD). **b** The SD-related negative DC shifts (mean ± stdev) for each experimental group. Black vertical arrows indicate KCl application or ES in aCSF, gray horizontal timelines depict the exposure to the experimental condition (HM or OGD). Red arrows highlight incomplete repolarization relative to pre-SD baseline. **c** The maximum amplitude of SD. **d** The duration of SD. **e** The slope of repolarization. *ES* bipolar electric field stimulation, *KCl* microinjection of 1 M KCl solution, *HM* hypo-osmotic medium, *OGD* oxygen–glucose deprivation. Data are given as mean ± stdev. Sample size (i.e. number of SD events analyzed) is indicated in each bar. One-way analysis of variance (ANOVA) followed by a Holm-Sidak post hoc test was used for statistical analysis. The level of significance is given as p < 0.001*** vs. KCl; p < 0.01^##^ and p < 0.001^###^ vs. ES; p < 0.001^$$$^ vs. HM

First, we compared the electrophysiological features of spontaneous SDs occurring in HM or during OGD to SDs evoked in normal artificial cerebrospinal fluid (aCSF). Prolonged SDs and incomplete direct current (DC) potential recovery were observed during OGD incubation and HM exposure compared to SDs evoked by KCl or electric stimulation (ES) in aCSF (33.95 ± 26.615 vs. 54.75 ± 26.72 vs. 195.65 ± 117.05 vs. 112.14 ± 88.39 s, KCl vs. ES vs. HM vs. OGD) (Fig. 1b, d). The incomplete DC potential recovery from SD was also reflected by the reduced slope of repolarization in the OGD and HM groups compared to aCSF (0.45 ± 0.27 vs. 0.50 ± 0.24 vs. 0.30 ± 0.17 vs. 0.12 ± 0.10 vs. mV/s KCl vs. ES vs. HM vs. OGD) (Fig. 1e). On the other hand, the greatest SD amplitudes were measured during HM exposure and ES (− 7.09 ± 3.4 vs. − 15.03 ± 5.01 vs. − 17.59 ± 5.3 vs. − 7.64 ± 5.33 mV, KCl vs. ES vs. HM vs. OGD) (Fig. 1c).

### Severe osmotic stress and oxygen glucose deprivation enhance the area invaded by SD

Next, we set out to explore whether longer SDs also invade larger tissue area, indicative of a larger bulk of tissue at risk of injury. To address this question, we performed IOS imaging of the brain slices. IOS imaging was also suitable to calculate the rate of SD propagation and SD latency (i.e. the time between the onset of HM or OGD incubation and the appearance of the SD focus).

**Fig. 2:**
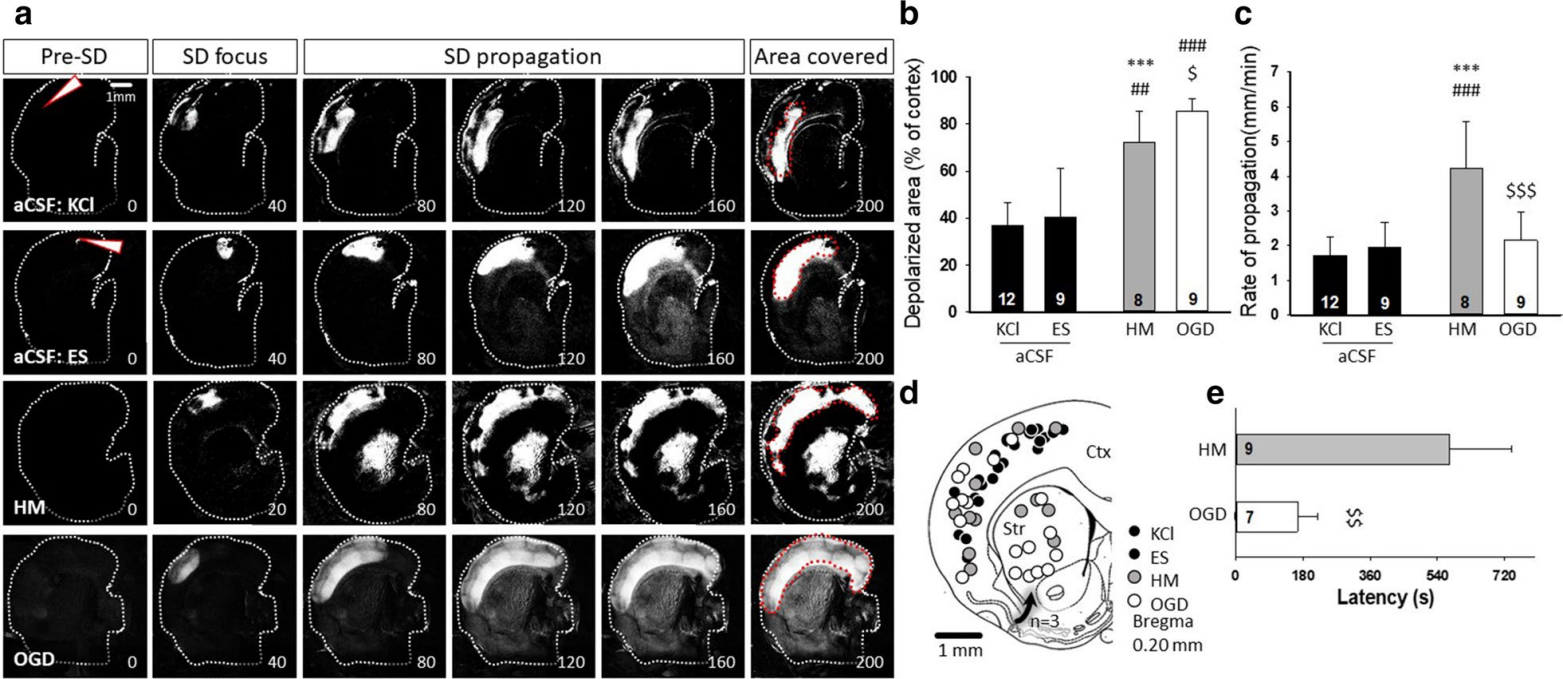
Intrinsic optical signal (IOS) imaging of spreading depolarizations. **a** Representative, background subtracted IOS image sequences demonstrate SD evolution across the experimental groups. Increased IOS intensity indicates SD. White arrowheads show the position of the KCl filled microcapillary or the stimulating electrode (ES) in the aCSF condition. Red dotted lines in “Area covered” delineate the maximal cortical area invaded by the SD. **b** The cortical area covered by SD relative to the total surface of the cortex. **c** The rate of SD propagation. **d** The localization of all SD foci observed in the cerebral cortex (Ctx) and the striatum (Str) in a schematic drawing. Some of the cortical SDs (n = 3) invaded the striatum at the ventral tip of the cortex, which is shown by the arrow. **e** The latency of spontaneous SDs with respect to HM or OGD exposure. *ES* bipolar electric field stimulation, *KCl* microinjection of 1 M KCl solution, *HM* hypo-osmotic medium, *OGD* oxygen–glucose deprivation, *Str* striatum, *Ctx* cerebral cortex. Data are given as mean ± stdev. Sample size (i.e. number of SD events analyzed) is indicated in each bar. A one-way analysis of variance (ANOVA) followed by a Holm–Sidak post hoc test (**b**, **c**) or a two-tailed T-test (**e**) was used for statistical analysis. The level of significance is given as p < 0.001*** vs. KCl; p < 0.01^##^ and p < 0.001^###^ vs. ES; p < 0.05^$^, p < 0.01^$$^ and p < 0.001^$$$^ vs. HM

The SD latency with respect to the onset of treatment was much longer in slices exposed to HM than to OGD (549.1 ± 164.59 vs. 165.75 ± 52.3 s, HM vs. OGD), while SD occurred within seconds after KCl or ES elicitation in aCSF (Fig. 2e). SDs during OGD or HM exposure propagated at a higher rate than SDs induced by KCl or ES in aCSF (1.7 ± 0.59 vs. 1.94 ± 0.73 vs. 4.24 ± 1.32 vs. 2.15 ± 0.83 mm/min, KCl vs. ES vs. HM vs. OGD) (Fig. 2c).

The tissue area representing the SD focus was enlarged during OGD or HM incubation compared to ES or KCl-evoked SDs in aCSF (1.37 ± 0.5 vs. 1.93 ± 0.74 vs. 2.64 ± 1.27 vs. 2.23 ± 0.82 % of the total cortical area, KCl vs. ES vs. HM vs. OGD) (Fig. 2a). SDs also invaded a significantly larger cortical area in the OGD and in the HM conditions compared to aCSF (36.72 ± 9.39 vs. 40.4 ± 20.66 vs. 71.98 ± 13.43 vs. 85.32 ± 5.32 % of total cortical area, KCl vs. ES vs. HM vs. OGD) (Fig. 2a, b). SDs initiated with a focal stimulus (KCl and ES) obviously evolved at the site of trigger delivery. During HM treatment or OGD, depolarization foci occurred in the parietal and temporal cortex (Fig. 2d).

Furthermore, SDs also occurred in the striatum during OGD in all slices examined (n = 9) (Fig. 2a, d). In HM, SD emerged in the striatum in 4 of 9 slices, and cortical SDs invaded the striatum from the ventral tip of the cortex in an additional 3 slices (Fig. 2d). The SDs with a striatal focus occurred invariably after the cortical SDs in these experiments, with a latency of 1–2 min with respect to the generation of the cortical SDs (34.85 ± 7.21 and 137.89 ± 59.39 s HM and OGD).

### Oxygen glucose deprivation and osmotic stress restrain cell survival after SD

**Fig. 3:**
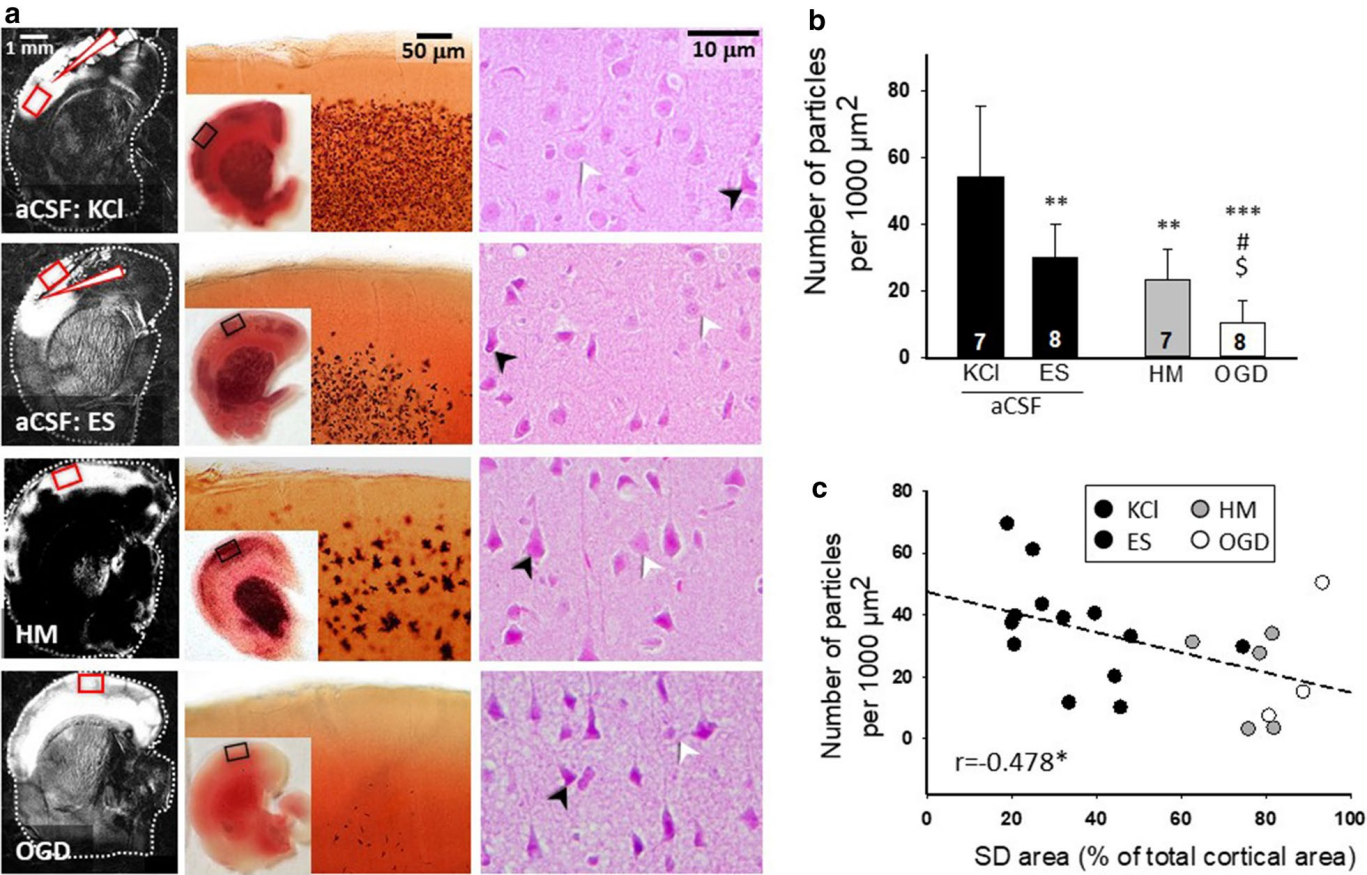
Histological analysis of SD-related tissue injury. **a** Representative background subtracted IOS images to the left show the surface of the cortex covered by the propagating SD (bright region). White arrowheads are pointing at the position of the site of SD elicitation with KCl or ES. In the middle, representative light microscopic images of brain slices stained with TTC after the passage of SDs (4–4 SDs were elicited in each slice with KCl or ES; a single SD occurred in response to HM or OGD) show macroscopic injury. Representative photomicrographs of hematoxylin–eosin-stained sections to the right demonstrate neuronal injury. Black arrowheads indicate injured neurons; white arrowheads show viable cells with intact nuclei in the somatosensory cortex. **b** The number of TTC-stained cellular compartments (i.e. particles). **c** Correlation between the number of particles and the cortical area bearing an SD as in Fig. 2b. Data are given as mean ± stdev. Sample size (i.e. number of slices analyzed) is indicated in each bar. *ES* bipolar electric field stimulation, *KCl* microinjection of 1 M KCl solution, *HM* hypo-osmotic medium, *OGD* oxygen–glucose deprivation. Statistical analysis relied on one-way analysis of variance (ANOVA) followed by a Holm-Sidak post hoc test (**b**), or one-tailed Pearson correlation analysis (**c**). The level of significance is given as p < 0.05* and p < 0.01** vs. KCl; p < 0.01 ^##^ and p < 0.001 ^###^ vs. ES; p < 0.05 ^$^ vs. HM

Finally, we moved on to analyze the histological outcome after the passage of SDs. 2,3,5-triphenyltetrazolium chloride (TTC) staining commonly used for the detection of lesion development [36–38] was applied to quantify the SD-related tissue injury. We accepted higher numbers of TTC-positive cellular compartments (i.e. particles) to indicate better tissue viability [31]. The SD-related TTC-positive particle loss was obvious after the single SD in HM or OGD (26.45 ± 10.64 and 11.44 ± 6.42 vs. 54.33 ± 21.21 per 1000 µm^2^, HM and OGD vs. KCl). Also, the repeated elicitation of SD with ES in aCSF caused a significant reduction in particle number (30.28 ± 9.84 vs. 54.33 ± 21.21 per 1000 µm^2^, ES vs. KCl) (Fig. 3a, b). Corresponding neuronal injury was observed in hematoxylin-eosin-stained sections: condensed, fragmented nuclei and vacuolization indicated pronounced neuronal damage after OGD and HM treatment (Fig. 3a). This was also seen to a lesser degree after recurrent SDs triggered with ES. In contrast, neurons were preserved and had a large, round nucleus with a prominent nucleolus after repeated, KCl evoked SDs (Fig. 3a). Finally, a linear negative correlation was found between the number of TTC-positive particles and the size of the cortical area engaged in an SD (Fig. 3c).

## Discussion

In the present study, our goal was the comparative evaluation of SD evolution in rat coronal brain slices exposed to osmotic or metabolic stress. SDs elicited with the focal application of 1 M KCl or bipolar electric stimulation in slices bathed in aCSF were used as reference. The application of OGD to study SD was justified by its widespread use to mimic severe global ischemia (e.g. cardiac arrest) or conditions prevalent in the ischemic core in focal cerebral ischemia [24, 39]. Of note, the less commonly implemented, partial metabolic challenge of submerged slices may be relevant to recapitulate ischemic penumbra conditions, although this model was reported not to give rise to spontaneous SDs [33]. Further, hypoxia at normal glucose levels has long been known to trigger SD, but this condition should not be confused with ischemia (i.e. ischemia is the restricted availability of oxygen, as well as glucose) [40]. To study the impact of osmotic stress on SD, we incubated the brain slices in HM, following a previously established approach to model cerebral edema [26, 31]. We experimented with HM solutions of decreasing Na^+^ content in our pilot experiments (120–100–80–60–40 mM, HM_120_–HM_40_, respectively), and selected here HM_60_ for systematic work, because incubation of the slices in HM_60_ reliably produced SD within 10 min after HM application. Higher Na^+^ concentration HM solutions in our pilot experiments (e.g. HM_100_) also caused SD occurrence, but at more variable and typically longer latency. The used Na^+^ concentration may occur spatio-temporally locked with SD in the in vivo brain, as the extracellular concentration of Na^+^ has been known to decrease from 140–150 mM down to 50–70 mM with SD [41]. The SD-linked osmotic stress, however, must be less pronounced, due to the compensatory accumulation of osmolites other than Na^+^ in the interstitium (e.g. K^+^ and glutamate) [41, 42]. Finally, as control for both OGD and HM, we elicited SD in aCSF with highly focused KCl pressure injection (picoliter volume), expected to be near the minimum conditions of SD elicitation [34], and the least invasive. This condition is thought to be relevant to model SD as it occurs in migraine with aura [43]. In other aCSF-incubated slices, electrical stimulation was applied for SD elicitation, due to its frequent application in a number of labs with the purpose to determine the threshold of SD elicitation [44, 45]. The otherwise standardized conditions (i.e. the level of the coronal brain slices, the region studied, the composition and temperature of the media, the type of the brain slice chamber and the standard approach of data acquisition) allowed the identification of SD features that occur typically in response to these well-defined and reliably reproducible osmotic or ischemic challenges.

Both OGD and HM gave rise to a spontaneous, long-lasting SD, at a short latency as previously shown (2.7–9.2 min) [35, 46]. These spontaneous events propagated over the entire cortical surface in the preparation. The brain slice was irresponsive to further stimulation after SD in OGD (Fig. 1), and displayed acute, extensive histological damage in the path of SD (Fig. 3), therefore SD has been accepted as a terminal event in the cortex in OGD. These results agree with earlier findings [46, 47]. In contrast, the tissue repolarized from the SD in HM – albeit with a long delay (Fig. 1)—confirming previous observations [35, 48]. The partial electrophysiological regeneration was supported by the propensity of the tissue to bear subsequent SDs either spontaneously occurring in HM (Fig. 1) or triggered with transient anoxia in HM [31]. In line with the functional data, the first SD in HM caused cellular injury in the cortex engaged in SD, but the damage was less severe than that seen after SD in OGD (Fig. 3). SD evoked by OGD sustained for 15–20 min here was associated with extensive injury in all layers of the cortex as evidenced by TTC staining (Fig. 3a), and in line with previous reports [46, 47]. Yet, some cortical neurons (e.g. in layer 4) may survive shorter OGD (about 10 min) followed by reperfusion with normal aCSF, and not reach a commitment point to neuronal death, as shown by patch clamp recordings [49]. Also, subcortical structures, such as neuron populations in the hypothalamus and the brain stem may be more resistant to OGD than the cortex [47, 50].

In the control conditions here (KCl or ES), a train of 4–5 short-lasting SDs could be elicited at an interval of 10–12 min in agreement with previous reports [32], which demonstrates the sustained viability of the tissue after the passage of SD under physiological conditions. Of the two control conditions here, focal low volume KCl application with pressure injection caused no detectable histological degeneration in the brain slice. However, ES-triggered SDs were accompanied with obvious damage to the tissue. Although not documented systematically, most SD researchers agree that electric stimulation with a charge sufficient to trigger SD leaves focal necrotic tissue damage behind at the electrode contact point [51]. This is also substantiated by the need to reposition the stimulating electrode between SDs evoked to estimate the electric SD threshold reliably [45]. Collectively, our results here present three stages of the SD continuum [51]: (i) apparently harmless SD in intact tissue followed by sufficient recovery, (ii) injurious SD with poor recovery emerging under osmotic stress, and (iii) terminal and detrimental SD in oxygen and glucose deprived tissue.

The distinct features of the negative DC shift and IOS signature of SD may disclose some mechanistic elements of SD evolution typical for the homeostatic conditions. For example, the slower rate of repolarization in HM and OGD as seen here (Fig. 2) is thought to correspond to the dysfunctional re-uptake of K^+^ or glutamate. The shortage of ATP in OGD causes the failure of the neuronal and astrocytic Na^+^/K^+^ ATP-ase, which hampers the uptake of K^+^ accumulated with the SD wave front in the extracellular space [5, 14]. Although osmotic stress could also attenuate the enzymatic activity of the Na^+^/K^+^ ATP-ase [52], HM exposure has been shown to induce prominent astrocyte swelling [31, 53] and to disrupt the astrocyte buffer capacity, reflected by a progressive accumulation of glutamate, for example [31]. Astrocytic K^+^ and glutamate re-uptake has been implicated in the repolarization after SD [54]. Taken together, we propose that the plausible cause of slower repolarization and prolonged SD duration in HM was the injured glial K^+^ clearance and glutamate transport. The failure of the Na^+^/K^+^ ATP-ase and the exhaustion of the astroglial buffer capacity are also suspected to contribute to the remarkably greater area engaged in SD propagation in HM and OGD compared to aCSF (Fig. 2).

SD in HM stood out from the other experimental conditions with its high rate of propagation and DC shift amplitude (Fig. 2). A recent study of ours has conducted extensive pharmacological manipulations and electrophysiological measurements to understand the mechanistic background of SD evolution in HM [31]. We have demonstrated augmented glutamate accumulation, a profound increment of evoked field potential amplitude and decreased electric threshold of SD elicitation in HM [31], which appear to be consistent with the enhanced neuronal excitability and intensified synaptic activity under osmotic stress [35, 55]. In addition, the shrinkage of the extracellular space in HM [26] may also account for the accelerated propagation rate of SD.

## Conclusions and perspectives

In summary, we show here that under otherwise standardized experimental conditions, SD is most pronounced in HM, in which condition the metabolic substrate supply is unlimited, but the tissue is hyper excitable due to cellular swelling and impaired glutamate clearance. In contrast with OGD, SD propagating under hypo-osmotic condition is not terminal, yet it is associated with irreversible tissue injury. This in vitro study offers a new perspective by the direct comparison of these two states, which must co-exist and intertwine in acute brain injury.

Finally, in vitro models, as presented here, offer an opportunity to screen the neuroprotective potential of therapeutically promising drug candidates, before testing them in in vivo systems. The pharmacological modulation of neurophysiological events that take place in the first minutes after acute brain injury may not seem clinically relevant. Yet, it must be realized that with the maturation of the injury, SDs become recurrent and at least some of them originating at the peri-infarct zone share basic features with the initial SD [1]. Also, to substantiate the value of OGD or HM brain slice studies: the pharmacological delay of SD onset in the OGD model proved to be a first step to later identify pertinent drug effect achieved by targeting sigma1 receptors [56, 57]. Therefore, experimentation in these simple model systems is expected to impart valuable understanding of SD pathophysiology, effectively complement in vivo work, and identify potential sites for intervention.

## Methods

 The experimental procedures were approved by the National Food Chain Safety and Animal Health Directorate of Csongrád-Csanád County, Hungary. The procedures were performed according to the guidelines of the Scientific Committee of Animal Experimentation of the Hungarian Academy of Sciences [updated Law and Regulations on Animal Protection: 40/2013. (II. 14.) Gov. of Hungary], following the EU Directive 2010/63/EU on the protection of animals used for scientific purposes, and reported in compliance with the ARRIVE guidelines.

Young adult, male Sprague-Dawley and Wistar rats (body weight: 250 g; n = 51) were used in this study. Animals were acquired from the Central Animal House of Biological Research Center, Szeged (Charles River Laboratories breed) and the animal house of the Department of Pharmacodynamics and Biopharmacy, University of Szeged (Charles River Laboratories breed). Standard rodent chow and tap water were supplied *ad libitum*. The animals were housed under constant temperature, humidity, and lighting conditions (23 °C, 12:12 h light/dark cycle, lights on at 7 a.m.).

### Live brain slice preparations

The procedures of live brain slice preparation were identical to those reported earlier [30]. The brains of the rats were removed under deep anesthesia (4–5 % isoflurane in N_2_O: O_2_; 2:1). Coronal brain slices (350 μm) anterior to bregma were cut with a vibrating blade microtome (Leica VT1000S, Leica, Germany), and collected in ice-cold aCSF (composition of aCSF in mM concentrations: 130 NaCl, 3.5 KCl, 1 NaH_2_PO_4_, 24 NaHCO_3_, 1 CaCl_2_, 3 MgSO_4_ and 10 dglucose). Four to six slices were transferred to an incubation chamber filled with carbogenated aCSF (difference in composition in mM concentrations: 3 CaCl_2_ and 1.5 MgSO_4_) and kept at room temperature (~ 20–22 °C). Individual slices were placed in an interface type recording tissue chamber (Brain Slice Chamber BSC1, Scientific Systems Design Inc., Ontario, Canada) and continuously perfused with carbogenated aCSF at a rate of 2.5 ml/min. Chamber temperature was kept at 32 °C using a dedicated proportional temperature controller unit (PTC03, Scientific Systems Design Inc., Ontario, Canada) [30].

### Local field potential recordings

LFP filtered in DC mode (< 1 Hz) was acquired via a glass capillary microelectrode (1–3 MΩ) filled with 150 mM NaCl and 1 mM HEPES. The microelectrode was inserted into the 3rd cortical layer, and an Ag/AgCl reference electrode was positioned in the recording chamber. The glass capillary microelectrode was connected to a custom-made dual-channel electrometer (including AD549LH, Analog Devices, Norwood, MA, USA), and the signal was fed to dedicated differential amplifiers and associated filter modules (NL106 and NL125, NeuroLog System, Digitimer Ltd., United Kingdom). The recorded analogue signal was converted to digital signal and displayed live using an Acqknowledge environment (MP 150, Biopac Systems, Inc) at a sampling frequency of 1 kHz [30]. DC potential traces confirmed the occurrence of SD events. Further, the DC potential recordings were used off-line to determine the amplitude, duration at half amplitude and the slope of depolarization and repolarization of SDs.

### Intrinsic optical signal imaging

For IOS imaging, slices were illuminated by a halogen lamp (Volpi AG, Intralux 5100, Schlieren, Switzerland). Image sequences were captured at 1 Hz with a monochrome CCD camera (spatial resolution: 1024 × 1024 pixel, Pantera 1M30, DALSA, Gröbenzell, Germany) attached to a stereomicroscope (MZ12.5, Leica Microsystems, Wetzlar, Germany), yielding 6–10× magnification. Image sequences were analyzed off-line; the area of the SD focus, the total area covered by the propagating SD, and the propagation velocity of SDs were analyzed after contrast enhancement in Fiji.

### Methods of SD elicitation

#### KCl injection

SDs were evoked by pressure injection of 1 M KCl (40 ms, 30 psi, approximately 150 picoliter) via a glass micropipette, using a picospritzer (40 ms, 30 psi, Picospritzer III, Parker Hannifin, Hollis, USA). The micropipette was lowered into the 3rd cortical layer at a distance of 500 μm from the recording electrode. Four-to-five successive SDs were initiated in each slice at 10–12 min intervals (n = 38).

#### Electric stimulation

In order to elicit SD with ES, a concentric bipolar needle electrode (WPI, Sarasota, FL, USA) was positioned onto brain slices, at a distance of approximately 800–1000 μm from the DC potential recording electrode. The elicitation of SD followed previously established principles [45]. The stimulating electrode was connected to an opto-coupled stimulus isolator with constant current output (NL 800, Digitimer Ltd., United Kingdom), a pulse generator (NL301), a width-delay panel (NL405), and a pulse buffer (NL510), which enabled the adjustment of the duration of the stimuli at will. Stimulation was implemented with single, cathodal constant current pulses. The charge delivered was increased stepwise until SD was initiated, quantified as Q [µC] = I [mA] × t [ms], and ranged between 50–100 µC. Four-to-five consecutive SDs were initiated in each slice at 10–12 min intervals to allow full recovery between events (n = 51).

#### Hypo‐osmotic challenge

Slices (n = 31) were exposed to HM, which were prepared by reducing the NaCl concentration of aCSF from the regular 130 mM to 60 mM, while other components and the pH of the medium were unaltered. Electrophysiological recording or IOS imaging was initiated during normal aCSF incubation, 10–15 min before switching to HM superfusion, and pursued 15–20 min into HM incubation. SD occurred spontaneously in response to osmotic stress.

#### Oxygen–glucose deprivation

OGD was induced by super-fusion of glucose-free aCSF (glucose was substituted with sucrose at equimolar concentration) on slices (n = 40), and the solution was gassed with 95 % N_2_ + 5 % CO_2_. Electrophysiological recording or IOS imaging was initiated during normal aCSF incubation, 10–15 min before OGD onset, and pursued for 15–20 min after OGD onset. OGD invariably gave rise to an SD event. In selected slices, aCSF incubation was resumed after OGD for 5 min, and OGD was imposed again for 30 min, to test whether a subsequent SD event could generate (Fig. 1).

### Histology

#### TTC staining

The size of the ischemic lesion was determined by TTC staining. The brain slices (n = 30) were incubated in a 2 % solution of TTC in 0.1 M phosphate buffered saline (PBS) for 20 min at room temperature. The sections were subsequently immersed and stored in 4 % paraformaldehyde for 24 h. The stained sections were then mounted on microscope slides and coverslipped with glycerol. Images were taken of the cortex with Nikon-DS Fi3 camera attached to a Leica DM 2000 Led light microscope (Leica Microsystems GmbH, Germany) at 10× magnification.

#### Hematoxylin–eosin staining

For proper visualization of cell damage, representative TTC stained slices were rinsed with PBS and cryoprotected in 30 % sucrose in PBS. Coronal, 10-µm-thick frozen sections were cut with a freezing microtome (Leica CM 1860 UV, Leica, Germany). The sections were stained with hematoxylin (Sigma-Aldrich, USA) for 25 s, rinsed with distilled water, then stained with eosin (Sigma-Aldrich, USA) for 15 s, rinsed with distilled water, dehydrated, and coverslipped with Eukit® (Merck, USA). The sections were examined with optical microscopy; photomicrographs at 40× magnification were taken with a Nikon-DS Fi3 camera attached to a Leica DM 2000 Led light microscope (Leica Microsystems GmbH, Germany).

### Data analysis

LFP traces were stored using a personal computer equipped with a dedicated software (AcqKnowledge 4.2 for MP 150, Biopac Systems, Inc., USA). Data analysis was conducted offline and was assisted by the inbuilt tools of AcqKnowledge 4.2 software.

Spatial features of SDs were measured in image sequences after background subtraction and manual thresholding in Fiji. The area of the SD focus and the total area covered by the propagating SD were expressed relative to the full surface area of the cortex in the brain slice. The calculation of the propagation velocity was aided by the IOS signal and was more accurate due to the perceptible direction of SD propagation. SD events with amplitude < 3 mV or those where the post-SD DC signal remained level were excluded from data analysis.

To characterize SD events, the amplitude, the slope of depolarization and repolarization, and the duration of SD were quantitated in the LFP traces filtered in DC mode. These variables are conventionally taken as a measure of the propensity of the tissue to bear SD (e.g. slower depolarization or smaller amplitude disclose lower SD susceptibility) [11, 58, 59], and to predict the injurious potential of SD (the duration of SD or the cumulative duration of recurrent SDs correlate with the degree of ischemic injury) [16, 60]. The duration of SD may also disclose the severity of the ongoing injurious condition. As such, SD becomes remarkably prolonged or terminal when the metabolic crisis in the tissue is severe [14].

After binary conversion, TTC stained images were manually thresholded, particles were measured with the inbuilt “analyze particles” function of FIJI and expressed as % area of baseline.

Quantitative data are given as mean ± standard deviation (stdev). Statistical analysis was conducted with the software SigmaPlot 13.0 (Systat Software, Inc. San Jose, USA). Data sets were evaluated by two-tailed T-test or ANOVA followed by a Holm-Sidak post hoc test, or one-tailed Pearson correlation. Levels of significance were set at p < 0.05.

## Data Availability

The datasets used and/or analyzed during the current study are available from the corresponding authors on reasonable request.

## Abbreviations

aCSF: Artificial cerebrospinal fluid
ANOVA: One-way analysis of variance
CBF: Cerebral blood flow
DC: Direct current
ES: Electric stimulation
HM: Hypo-osmotic medium
IOS: Intrinsic optical signal
LFP: Local field potential
OGD: Oxygen glucose deprivation
PBS: Phosphate buffered saline
SD: Spreading depolarization
SUDEP: Sudden unexpected death in epilepsy
TTC: 2,3,5-Triphenyltetrazolium chloride
